# Ocular Manifestations of Behçet’s Disease: An Update on Diagnostic Challenges and Disease Management

**DOI:** 10.3390/jcm10215174

**Published:** 2021-11-05

**Authors:** Hanna Zając, Anna Turno-Kręcicka

**Affiliations:** 1 Department of Ophthalmology, University Clinical Hospital in Wroclaw, 50-556 Wroclaw, Poland; 2 Department of Ophthalmology, Wroclaw Medical University, 50-556 Wroclaw, Poland

**Keywords:** Behçet’s disease, ocular Behçet, uveitis, management of Behçet’s disease

## Abstract

Behçet’s disease is a systemic vasculitis that affects multiple organs. The most common manifestations are oral and genital ulcerations and recurrent uveitis. Uveitis can be an initial symptom in 10–20% of cases and leads to blindness in 16–25% of patients. The management of this disease is evolving due to the clinical phenotypes recently described in the literature and increasing focus on the detection of subclinical inflammation to enable correct therapeutic decisions. The first line treatment is azathioprine, followed by various immunosuppressive and biological agents as alternatives in severe or refractory cases. This review summarizes scientific articles about the etiology of, diagnostic tools for and treatment of the ocular manifestations of Behçet’s disease available in the PubMed database from 1 January 2016 to 1 May 2021. A multidisciplinary approach is necessary to effectively prevent permanent damage and thus improve the life quality of the patients. Therefore, it is crucial to raise awareness of the common clusters of symptoms, use of modern imaging methods, such as ocular computed tomography and fluorescein angiography, and novelty treatment algorithms to enable early diagnosis and appropriate management.

## 1. Introduction

Behçet’s disease (BD) is a chronic, multi-system, relapsing vasculitis of unknown etiology. It affects both genders at a different ratio depending on the region, but mostly is predominant in male [1]. The typical time of onset is between the second and the fourth decade of life. Nevertheless, it may also start in childhood (4–26% incidence) [2] or at an older age. It is mainly sporadic; however, there have been some familial cases, suggesting a complex inheritance model of the disease. Mucocutaneous aphthous lesions in the oral cavity and genital area and recurrent uveitis are the most common symptoms. Death rate is approximately 5% in 5–10 years [3], especially due to cardiovascular or central nervous system (CNS) complications. The prognosis is poorer for male patients with a younger age of onset [1,4,5] and multiple manifestations at the time of diagnosis [2,3,6,7].

BD is one of the most severe causes of noninfectious uveitis and occurs in up to 50–90% of patients [3,8,9,10]. Behçet’s uveitis (BU) can lead to blindness that affects 16–25% [10] of patients within 5–10 years after onset [11]. BU develops usually after 2–3 years [8]; however, there may be an initial presentation of this systemic disease (10–20%) [3,8,9].

The highest prevalence of BD is reported in the Middle East and East Asia. The exact prevalence in Poland is unknown; however, it is believed to be underdiagnosed [12] (Table 1).

According to a genome-wide association study Human Leukocyte Antigen, B51 (HLA-B51) is the strongest genetic/endogenous factor in BD. Between 40% and 80% of patients with BD have HLA-B51, whereas it is present only in 10–30% of healthy controls. For this reason its detection is valuable information, even though it is not a part of the official diagnostic criteria [16]. Moreover, patients with HLA-B51 are more prone to developing ocular manifestations of BD. A strong correlation is observed in regions towards the east, along the ancient Silk Road in Eurasia; however, it is not statistically significant in Europe [16,17]. HLA-A26 carriers have a higher risk of developing iridocyclitis and retinochoroiditis, although its impact is more prominent for men [18].

The results of a nationwide survey in Germany corroborate the role of genetic background of BD. The prevalence of BD among patients of Turkish origin living in Germany was similar to that reported from Western Turkey (20–80/100,000), while the prevalence in Germany (0.3/100,000) was comparable to other North European regions [15]. Population-based prevalence studies in France have found that immigrants of North African and Asian ancestry have a similar prevalence of BD to the countries of their origin. Moreover, the prevalence of BD is not influenced by the age of the individual at the time of immigration, which strongly supports the hereditary basis of the disease [19]. Kötter et al. compared patients with BD of Turkish origin living in Germany with those living in Turkey, and with German patients. The authors did not reveal any differences that would support the influence of ethnicity on the expression of BD, which stands in contrast to the previously mentioned studies. They proposed that the higher frequency of ocular manifestations and a higher male to female ratio in Turkish patients living in Germany compared to those living in Turkey may be due to environmental factors [20]. A study by Shahram et al. comparing a selected group of US patients with Iranian and Turkish patients revealed some interesting findings. Even though there was a higher proportion of women in the US group, US patients had more severe presentations of the disease than the Iranian and Turkish patients. The American patients were more often diagnosed with multi-organ manifestations and were less prone to developing ocular vasculitis. On the contrary, the Iranian patients were more likely to present with a single-organ disease and were at higher risk for ocular vasculitis. This may be caused by milder cases being underdiagnosed in the United States [14]. The authors believe that ocular and extra-ocular BD may follow independently regulated pathogenesis, as well as differing according to the geographical and genetic background of the patient [7,14].

A retrospective study by Hussein et al. conducted on an Egyptian cohort (249 patients) revealed a total of 51% of cases with ocular involvement. Patients presented with anterior (59%) and posterior uveitis (74.8%), panuveitis (33.8%), retinal vasculitis (31.4%), papillitis (7.8%) and chorioretinitis (54.3%), macular edema (9.4%) and secondary retinal detachment (7.8%). A total of 74.01% of cases were classified as having a vision-threatening disease (VTD). It has been found that the absence of the systemic criteria (genital ulcers, systemic vasculitis and, maybe oral ulcers as well) may mean that the eye is at higher risk for the development of VTD [21].

Multidisciplinary collaboration is essential for treatment, as it depends on the current organ involvement and factors, such as age, gender, type and severity of symptoms and disease duration. The goal is to develop targeted therapies, prevent relapses and suppress inflammation [7].

The aim of this review is to focus on the management of the ocular manifestation of BD that are described in the literature.

## 2. Materials and Methods

This review was prepared by using the database of PubMed. Articles from peer-reviewed journals published from 1 January 2016 to 1 May 2021 were chosen using the following search words: ocular Behçet, and uveitis Behçet. This was followed by manual searches based on articles cited in the texts of other articles. We included articles contributing to the topics of the epidemiology, diagnosis, and treatment of ocular manifestations of Behçet’s disease. Clinical studies were selected if they were randomized controlled trials, single- or double-blind, or interventions with pharmacological therapy compared to placebo or some other pharmacological agents or unrandomized studies with valuable remarks for future investigation. Abstracts were used in the case of non-English articles. All of the figures used in the articles have been obtained from the authors’ clinic.

## 3. Results

### 3.1. Etiopathogenesis

There are probably several pathways of pathogenesis—there is an interplay of genetic susceptibility factors with unbalanced immune homeostasis. Positive responses to immunosuppressive agents, the involvement of autoantigens, and antigen-specific T-cells suggest an autoimmune background. Seemingly unprovoked episodes of inflammation may indicate an autoinflammatory origin [22,23]. It shares some features of a spondyloarthropathy, based on the association with HLA-B51, epistatic endoplasmic reticulum aminopeptidase 1 (ERAP-1) interactions, increased T helper (Th) 17 response, and neutrophilic involvement [22,23]. However, infectious agents have been proposed as a trigger of BD development [2]. Cross reaction of microbial antigens and human proteins may lead to a pathological immune response in genetically predisposed individuals. Herpes simplex virus 1 and bacteria of the Streptococcus species (among others, *Streptococcus sanguinis* and *Streptococcus pyogenes*) has shown evidence of the highest correlation with BD [22,23]. It is speculated that oral microbial flora plays a role in the pathogenesis of BD, as it usually starts in the oral mucosa and tends to flare up after dental and surgical procedures in the oral cavity. It has been reported that BD patients have less diverse salivary and gut microbial flora in comparison to healthy controls [23].

Retinal pericytes, which may influence the intravascular immunity, are of the same origin as the CNS pericytes and may function differently from the peripheral vascular pericytes. Hussein et al. suggest the possibility of having two immunological variants of BD (with central and peripheral impact), which corresponds with the findings of the abovementioned study by Shahram et al. [14,21].

### 3.2. Diagnostic Criteria

The most common classification criteria for the diagnosis are International Criteria for Behçet’s Disease (ICBD) and the International Study Group (ISG) criteria. In a study comparing the ICBD 2006, revised ICBD 2010, ISG criteria and revised Japanese criteria, it was found that the ICBD 2010 had the highest sensitivity (98.83%), negative predictive value (98.48%), diagnostic odds ratio (1645), and Youden’s index (0.94), and the lowest negative likelihood ratio (0.01) [24].

#### ICBD Criteria

- Ocular lesions (uveitis, retinal vasculitis, chorioretinitis, papillitis)—two points;
- Oral aphthosis of at least three times/year—two points;
- Recurrent genital aphthosis—two points;
- Skin lesions (papulopustular rash, erythema nodosum)—one point;
- CNS lesions (parenchymal CNS involvement, venous sinus thrombosis)—one point;
- Vascular manifestations (venous thromboembolism, superficial thrombophlebitis, arterial thrombosis, aneurysm—especially aortic and pulmonary)—one point;
- The positive pathergy test—one point;
- A patient scoring ≥ 4 points is classified as having BD [24].

### 3.3. Extra-Ocular Manifestations

Oral aphthae are often a presenting sign of BD. They are round, painful ulcerations with a diameter of 2–15 mm. They last for 7–10 days and heal with no scarring, unless large. Painful genital ulcerations appear mostly on the scrotum and labia and are similar to the oral ulcerations. They are usually bigger, deeper and are more likely to heal with scarring.

The most common skin lesions are recurrent erythema nodosum, papulopustular lesions on upper torso and extremities and folliculitis-like lesions. Nearly 40% [6] of the patients present with pathergy positive BD, although it is not pathognomonic for BD and may not be present when the patient is already under systemic immunosuppressive treatment [7,12,17].

Arthritis develops in up to 50% [6] of patients. It is usually monoarticular, non-erosive, and self-limiting, with attacks lasting a few weeks. Intestinal BD manifests as ulcers of the esophagus, stomach, and intestines [6,7,25].

Vascular involvement occurs in 25% [6] of patients. It varies from the superficial vein to the superior/inferior vena cava thrombosis, vessel occlusion, and arterial aneurysms. Cardiac complications include pericarditis, granulomatous endocarditis, myocarditis, coronary arteritis, myocardial fibrosis, and intracardiac thrombosis. Pulmonary artery aneurysm is one of the most lethal complications and is considered to be almost pathognomonic for BD [6,7,25].

Neuro-Behçet’s Syndrome occurs in 5–10% [7,25] of BD patients, mostly (75–80%) [7] affecting the CNS. Neurological involvement poses a great threat to the patient, as lesions of the white matter of the brain and brainstem may lead to motor dysfunction, cognitive and behavior changes and stroke. Neuroophthalmological findings, such as cranial nerve palsies, papillitis and papilledema may occur due to the thrombosis of the venous sinuses [6,7,25].

### 3.4. Ocular Manifestations

Behçet’s uveitis is defined as a chronic relapsing bilateral nongranulomatous panuveitis and retinal vasculitis [9]. BU can affect both the anterior and the posterior segment of the eye; however, panuveitis is the most frequent presentation [26]. Only 10% of patients—most of whom are women—present with an isolated anterior uveitis [25,27].

**Figure 1:**
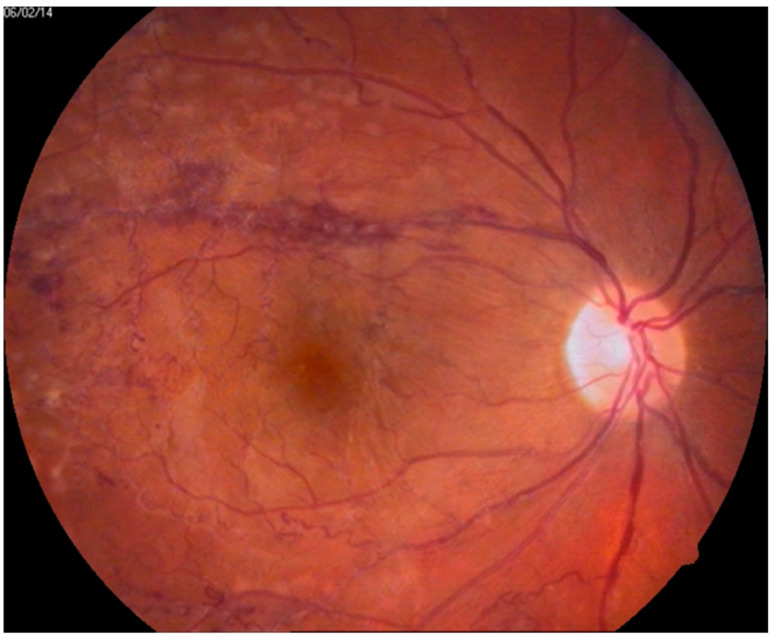
Color eye fundus photographs of patients with Behçet’s Disease. Fundus presents with vascular changes of the superior arcade, widened, irregular and tortuous veins, intraretinal hemorrhages and premacular inflammatory fibrosis.

Occlusive necrotizing retinal vasculitis is a critical component of BU [28]. In fundoscopy, periphlebitis has the form of perivascular diffuse white haziness [9] and may be accompanied by periarteriolitis, which never occurs alone [2,28]. Thrombotic incidents are typically bilateral inflammatory branch retinal vascular occlusions. They lead to arteriolar attenuation and retinal non-perfusion, followed by retinal neovascularization [29], which is a possible source of retinal hemorrhage [2] (Figure 1) or even hemorrhagic periphlebitis [9]. After the resolution of the acute inflammatory process, the eye fundus may present with sheathed ghost vessels [9]. Frosted branch angiitis, with or without neuroretinitis, is another possible manifestation [9].

Breakage of the blood–retinal barrier due to inflammation results in perifoveal capillary leakage that causes cystoid macular edema (CME) and further macular structural changes. Vascular leakage is observed within the optic disc, peripheral retina, and macula [10,14,26,29]. CME is diagnosed in up to 60% of BU cases [30] and poses a potential threat of vision loss [2,9,26,28,29,31,32].

Vitreous haze is a sign of an active inflammation in the posterior pole. Vitritis is most prominent at the beginning of the attack and dissolves gradually [2,27], causing a relapsing visual blurring [9]. A pathognomonic sign for BU is inferior, pearl-like peripheral inflammatory precipitates organized in a linear pattern after 4–7 days from the uveitis onset that disappear without any sequel within weeks [2,32].

Hypopyon is a poor prognostic factor [7] that has been long considered a hallmark of BU, although nowadays is known to be a rather nonspecific sign [1,9]. It is present in only 30–40% of cases [29]. A characteristic feature of BU is a lack of fibrinous exudate in the anterior chamber. Therefore, the hypopyon is non-sticky and can move freely with gravity [27,29,33]. Even though spontaneous remission within days to weeks is a known natural course of BU [32], it is unlikely to be observed in the clinical practice, as patients almost always receive intense treatment [27,33]. Anterior segment inflammation usually presents with mild or no ciliary injection, diffuse endothelial dusting and normal or low intraocular pressure [2,9]. Absence of mutton-fat keratic precipitates, chronic cells and chronic high-grade flare is typical for BU [9].

Severe BU results in retinal atrophy with clear vitreous, optic and macular atrophy, diffuse atrophy and gliosis of the retina with sheathed and attenuated cord-like white retinal vessels, which can mimic retinitis pigmentosa [9].

### 3.5. Diagnostic Tools

#### 3.5.1. Spectral Domain Optical Coherence Tomography (SD OCT)

SD OCT can be a screening tool for posterior involvement in Behçet’s uveitis. Superficial retinal infiltrates or their sequels are the most common finding in the fundus during active inflammation. They present as white patches that do not obscure underlying vessels and disappear within days with no scarring [9]. In the SD OCT they present as a focal, hyper-reflective thickening of the retina, blurring of the inner retinal layers and optical shadowing without thickening of the underlying choroid. The retinal pigment epithelium is not disrupted [2,27]. A wedge-shaped retinal nerve fiber layer defect and thinning are possible sequels of the retinal infiltrates [2,9,32]. Localized vitreous condensation over the inflamed optic disc characteristically forms a “smoking volcano” shape in SD OCT [28], which can be used to observe the regression of the vitreous haze over the optic nerve in neuroretinitis [2,27].

The mean central macular thickness (CMT) and macular volume (MV) are increased in eyes with Behçet’s posterior uveitis and panuveitis, even in the absence of macular edema [28]. Therefore, the use of fluorescein angiography (FA) may be targeted for cases with the highest suspicion of posterior uveitis [28]; however, it still remains the gold standard in the monitoring of BU, as OCT does not visualize the retinal vasculature [2,27,32]. Kang et al. have reported a decrease in CMT and MV after 2–3 months of treatment, which suggests the value of SD OCT as an additional monitoring tool [28]. Enhanced deep imaging OCT (EDI OCT) is another complementary examination for the detection of the subclinical ocular and systemic inflammation that can be indicated by increased choroidal thickness in an eye without clinically active uveitis [34].

Gürlü et al. performed a study comparing OCT findings in BD patients in uveitis remission and healthy individuals. A significant decrease of central macular thickness (CMT), decreased parafoveal Ganglion Cell Complex (GCC) and increased perifoveal GCC thickness was found in the remission group. CMT decreases with the number of uveitis attacks. The authors speculated that the GCC perifoveal thickness increases due to the occlusive changes in the superficial capillaries and local limited ischemia and may decrease later as a consequence of cell loss, which had already happened in the parafoveal area [26].

Optical coherence tomography angiography (OCTA) generates three-dimensional images of ocular fundus vasculature. Emre et al. recommend this method to visualize microvascular changes in patients with BU and other forms of uveitis. They found areas of hypoperfusion and nonperfusion in both deep capillary plexus (DCP) and superficial capillary plexus (SCP); however changes in DCP were more prominent than those in SCP. Moreover, the capillary vessel density was significantly lower in patients with BU than in the control group, and foveal avascular zone (FAZ) was reported to be larger in patients with BU. According to the authors, OCTA is superior to fluorescein angiography (FA) in terms of evaluating FAZ [35].

Zarei et al. claim peripapillary OCT to be a quantitative non-invasive method of assessing ocular inflammation that can serve as an alternative to FA, reducing the sessions of FA acquisition. Although FA remains the modality of choice to determine the extent of retinal vasculitis in BD, its strong dependence on the examiner may interfere with a standardized interpretation and comparison. For each micron increase in peripapillary retinal nerve fiber layer thickness (PNFLT), there was an 0.008 increase in logMAR and thus a deterioration of visual acuity. The authors draw attention to the fact that a conventional format of the OCT data output is set to detect “thinning”, not “thickening”, and therefore there is no color-coding to differentiate a thickened PNFL from a normal PNFL [36].

#### 3.5.2. Fluorescein Angiography

**Figure 2:**
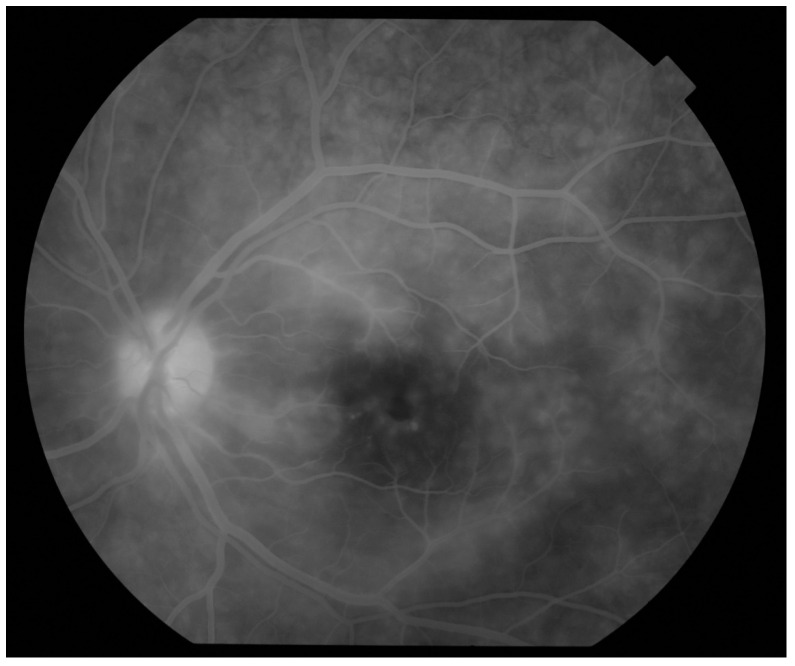
Fluorescein angiography of a patient with Behçet’s uveitis with cystoid macular edema, venous leakage, leakage from the cilioretinal artery and optic disc edema.

The advantage of the fluorescein angiography (FA) over angio-OCT is the ability of the former to visualize the whole fundus, while OCTA is limited to the posterior pole. Therefore, FA enables the detection of peripheral areas of insufficient perfusion that would benefit from laser photocoagulation [32]. In the FA, patients with occlusive and diffuse vasculitis present with fern-like peripheral leakage, optic disc and posterior pole leakage (Figure 2), engorgement and tortuosity of the veins and staining of the vessel walls [2,27,32]. A total vascular leakage score has been proposed to evaluate the course of uveitis, that is a sum of scores for the lesions at the disc, macula and peripheral retina in both eyes. The extent of leakage is described by points (0 = none, 1 = mild, 2 = moderate, 3 = severe) [29].

Chams et al. investigated a group of 50 patients (100 eyes) that had no apparent ocular signs in either biomicroscopy or fundoscopy. The study revealed that 44% of patients had leakage of fluorescein at the peripheral retina in both eyes in the FA, mostly from the final branches. Moreover, four of them presented leakage from the optic disc and one from the posterior pole. In the infrared autofluorescence of the fundus, 86% of patients presented with modified hypo- and hyper-autoreflectivity. This may be secondary to changes in the pigmentary cells and vascular modifications in the choroid. A total of 50% of patients presented with aberrant retinal vascular branching: either tortuous, straightened or with a vascular shunt [31]. FA may be useful for early diagnosis and the prompt introduction of treatment, as suppressing chronic subclinical inflammation is important to prevent complications [2,6,31,32].

Conventional fundus cameras can caption 30° to 60° view of the fundus at a time, while a recently introduced ultra-wide-field (UWF) imaging system provides 200° photographic, autoflourographic and angiographic view of the ocular fundus. UWF images may contribute to a more accurate detection of disease activity and appropriate disease management. In a prospective study comparing clinical decisions in cases of noninfectious retinal vasculitis, treatment was changed after 10% of visits based on clinical examination or standard fluorescein angiography, and after a further 24% of visits with UWF color photography and 51% of visits based on UWF fluorescein angiography [37].

#### 3.5.3. Other Diagnostic Methods

Laser flare-cell photometry (LFCP) is an objective method used to quantify flare and cell count in the anterior chamber (AC). Its use is limited because of a possible overestimation of cell count due to other particles in the AC, such as pigment discharge or red blood cells. The measurements are also unreliable in cases of very high grade of inflammatory cells or any particular matter in the AC [38]. Tugal-tutkun et al. investigated the use of LFCP in patients with BD. They reported no increase in flare in patients without ocular involvement, while it was significantly higher in patients during ocular attack or in remission. There was a significant correlation between FA leakage and AC flare. Moreover, the risk of recurrent uveitis attack was significantly higher in eyes with flare values > 6 photons/msec than in eyes with flare below this value. They claim that LFCP is a reliable method to monitor ocular inflammation in BD, which can decrease the need for FA and may be useful in clinical trials due to its quantitative outcomes [39].

Even though the indocyanine green angiography (ICGA) may present some nonspecific changes, dark dots representing choroidal granulomas are never found [9]. ICGA appears not to add further clinically relevant information about BD, other than differential diagnosis towards granulomatous anterior uveitis or choroiditis [9] and detection of central serous retinopathy as a side effect of a high-dose CS therapy [32].

Optic neuritis without intraocular inflammation is a part of neuro-BD [40]. It can result in visual acuity loss and impaired color vision. Mahgoub et al. have conducted visual evoked potential (VEP) examination in patients with BD with no recent ocular manifestation. They have reported a statistically significant positive correlation between p100 latencies and BD activity scores that decreased after steroid treatment for other active lesions, which may suggest subclinical visual pathway involvement [41]. Taking into consideration the point that ocular and neurological manifestations of BD are believed to coexist as a part of one phenotype [42], VEP may serve as a tool in the search of subclinical neurological involvement in ocular BD.

The chronic inflammatory process in BU has been reported to influence the cornea [43,44]. Cankaya and Kalayci have found an increase in the central corneal thickness and corneal resistance factor during uveitis attacks [43], whereas Ozbek-Uzman et al. reported a decrease in corneal thickness (CT) and corneal volume (CV) during an inactive period. CV and CT were significantly correlated with the frequency of previous uveitis attacks. The corneal changes were found to be independent from the location of uveitis [43]. These changes may be explained by endothelial pump and barrier dysfunction during the active period [44] and the stimulation of apoptosis and degradation of the fibrils by the inflammatory molecules present in the anterior chamber during remission [43].

A significant decrease of Tear Break-Up Time [45,46] and increased frequency of squamous conjunctival metaplasia and goblet cell loss [46] have been reported in patients with ocular BD. However, ocular BD has not been associated with quantitative Meibomian gland changes [46].

Behçet’s disease ocular attack score 24 (BOS24) is a novelty scoring system, designed to evaluate the activity of ocular inflammation in BU [47]. The BOS24 summarizes points given according to the following symptoms during an active uveitis episode: anterior chamber cells (0–4 points), vitreous opacity (0–4 points), peripheral fundus lesions (0–8 points), posterior pole lesions (0–4 points), subfoveal lesions (0–2 points) and optic disc lesions (0–2 points) [47]. The authors believe that the results of all of the attacks over five years (BOS24-5Y) are a more reliable characteristic of disease activity than only the frequency of relapses [47]. Keino H. consider this to be an objective and quantitative method and believe it to be a valuable tool to determine the timing of initiation and withdrawal of the treatment with anti-TNF agents [29].

### 3.6. Differential Diagnosis

The most important differential diagnosis to be considered are briefly summarized in Table 2.

### 3.7. Treatment

BD is a multidisciplinary entity, the division of which can be approached in different manners. Several clusters of BD manifestations have been identified, such as: “the mucocutaneous and articular phenotype”, “the extra-parenchymal neurological and peripheral vascular phenotype” and “the parenchymal neurological and ocular phenotype” [42,51]. Uveitis was not significantly correlated with neurological manifestations in a BD phenotype analysis in China; however, it was suggested that parenchymal involvement was rare across the race and ethnicity of the cohort [52]. Bettiol et al. suggest that BD treatment should be targeted at clusters of symptoms instead of focusing on each presentation separately [42].

Both the anterior and the posterior segment of the eye can be affected by inflammation; however controlling the posterior uveitis is the most crucial factor to preserve the best possible visual acuity [26]. There have been developed various therapy schemes of local, and systemic treatment that depend on the affected structures and severity of the disease [7].

Isolated anterior uveitis can be treated with topical corticosteroid (CS) drops at a high initial frequency [2,22], tapered to stop after 6–8 weeks [2,22]. Mydriatic and/or cycloplegic drops should be administered 2–3 times a day for 2–3 weeks [2]. In case of poor prognostic factors, systemic immunosuppressive treatment with AZA is indicated [7].

#### 3.7.1. Corticosteroids

Acute posterior uveitis exacerbations should be treated aggressively due to the potential threat of severe vision loss. The treatment of severe cases of the ocular and parenchymal neurological phenotype should be induced with high-dose corticosteroids (CSs), followed by gradual tapering over 3–6 months [42]. Acute posterior uveitis attack should be treated with intravenous pulse methyl prednisolone (IVPM) 250–1000 mg for 1–3 days, followed by oral 1 mg/kg/day with slow tapering—until the maintenance dose of ≤7.5 mg [49] is reached or with a high oral dose (1–1.5 mg/kg/day) [53,54]. IVPM are believed to prevent visual loss in acute phase of BU, and they are followed by less complications than a long period of therapy with high-dose CSs [54]. CSs should be always used together with immunosuppressive agents as a bridging therapy [2,7,25,27,54,55,56].

Intravitreal or periocular CS injections are suggested to be an effective adjuvant therapy in a unilateral disease, refractory CME, in patients with contraindications to systemic CSs or when an adequate response to the systemic therapy is not achieved [14,55]. According to the study of Yalcinbayir et al., in which a dexamethasone intravitreal implant (0.7 mg) was injected to the eyes with CME in BU, the highest visual gain was reached within the first two months following the injection, and 48% of eyes gained at least three lines of visual acuity [30]. However, possible complications, such as cataracts (7.4–36%), increased intraocular pressure (14.8–43%) and glaucoma (9%), should be taken into consideration [30]. It has been reported that a single-dose infliximab infusion is more efficient in suppressing acute episodes than intravenous or intravitreal CSs and may serve as an alternative [7,30].

#### 3.7.2. Immunosuppressive Treatment

European League Against Rheumatism (EULAR) have issued in 2018 an update of the recommendations for the treatment of BD. Azathioprine (AZA) and cyclosporine-A (CsA) have the highest level of evidence and strength of recommendation for patients with posterior uveitis [55].

Azathioprine is reported in the literature to be effective in the treatment of BU in the dose of p.o. 2–2.5 mg/kg per day [7,42]. Bettiol et al. consider it an adequate induction treatment for the ocular and parenchymal neurological phenotype [42]. AZA decreases the rate of hypopyon uveitis and new eye disease [7] and furthermore preserves visual acuity and prevents relapses [55]. AZA and IFN-alpha should not be combined due to the risk of myelosuppression [11].

Cyclosporin-A (CsA) (p.o. 5 mg/kg/day) is proven to decrease the frequency and severity of relapses in BU [7,55]. However, the use of CsA is contraindicated in the active neuro-BD [55] and therefore should not be implemented in the “parenchymal neurological and ocular phenotype” [42,57]. An increased prevalence of CNS manifestations has been reported in patients under this drug [58].

Bettiol et al. imply cyclophosphamide (CYC) (1 g/month for six months and then every two to three months) to be the third line treatment for the ocular and parenchymal phenotype [42].

Mycophenolate mofetil (500 mg–2 g/day) [5] and methotrexate (7.5–20 mg/week) [5] have been suggested to be alternative immunosuppressive options.

#### 3.7.3. Biological Treatment

According to EULAR reccomendations stated in 2018 biological treatment should be used as the second line treatment, as its efficacy was not supported by any randomized controlled study by the time of the update [55]. They outline that interferon-alpha, infliximab (IFX) and adalimumab (ADA) are preferred by some experts for the management of patients who are refractory to AZA and CsA [55].

**Figure 3:**
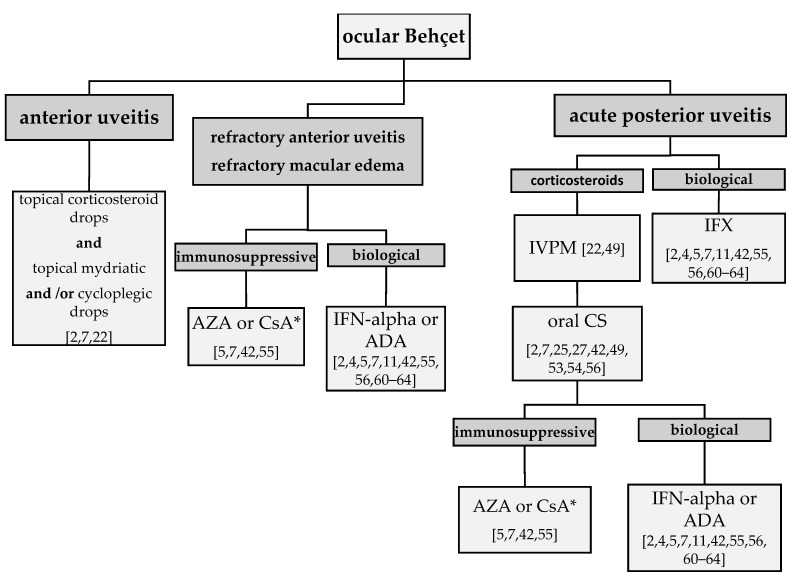
Proposed choice of therapeutic options, depending on the localization and severity of ocular inflammation. AZA—azathioprine; CsA—cyclosporine A (*)—NOT in the parenchymal neurological and ocular phenotype [42,57,58]; IFN-alpha—interferon alpha; ADA—adalimumab; IFX—infliximab; IVPM—intravenous pulse methyl prednisolone; CS—corticosteroid.

The Ocular Immunology and Uveitis Foundation have stated that BD with retinal involvement is an absolute indication for an early use of immunomodulatory therapy. They stress its importance in the cases of sight-threatening uveitis and for patients who are refractory to corticosteroids [59]. Interferon alpha (IFN-alpha) and anti-tumor necrosis factor (TNF) agents, such as infliximab (IFX) and adalimumab (ADA), are widely recommended as first or second-line treatment options for refractory and/or recurrent cases [2,7,42,55,56,60,61,62,63,64]. The choice of the immunomodulatory therapy depends on the severity of inflammation and on the time in which the drug provides therapeutic effect (Figure 3).

In a review by Thomas A.S., the use of ADA and IFX is indicated as the first-line therapy of uveitis in BD, whereas for most other noninfectious uveitis entities these drugs remain a second choice [62].

Levy-Clarke et al. have given a strong recommendation of a panel of experts based on an extensive review of literature from 2014 about the use of anti-TNF agents in the therapy of BD. They suggest that IFX and ADA are adequate for first- or second-line corticosteroid-sparing therapy with ocular BD. Moreover, IFX may be a first- or second-line treatment for acute exacerbations of pre-existing BD [65]. This corresponds with the algorithm of treatment of BD uveitis by Karadag et al. from 2020, that included IFX or IFN-alpha as first-line therapy for acute sight-threatening uveitis at presentation together with high-dose intravenous corticosteroids (CSs) [7]; however, they suggested only AZA and CsA as the first-line therapy for posterior uveitis or panuveitis together with oral CSs, whereas IFX, ADA or IFN-alpha were indicated in refractory and/or recurrent cases [7]. Bettiol et al. reported that increasing observational evidence supports the use of IFX and ADA as second-line therapy in both ocular- and neuro-BD [42].

IFX is administered intravenously in a loading dose of 3–5 mg/kg (0–2–6 weeks), followed by a maintenance dose every 4–8 weeks [4,7,59,66,67,68,69]. The optimization of the IFX therapy is recommended after at least 12 months, once the ocular remission has been achieved for 3–6 months. It can be introduced either by gradually prolonging the dosing intervals or by reducing the dose to 3 mg/kg every eight weeks and then prolonging the dosing intervals. Treatment should be discontinued once an interval of 12 weeks has been achieved in the absence of ocular inflammation. Therapy should be restarted in case of a relapse (5 mg/kg i.v. every eight weeks) [67]. According to Markomichelakis et al., intravenous IFX should always be considered in patients with panuveitis attack in BD. They noted a significantly faster decrease of the ocular inflammation after a single IFX infusion, compared to intravenous and intra-vitreal CSs. IFX was superior to CSs in the regression of cystoid macular oedema, retinal vasculitis, and retinitis; however the improvement of visual acuity was comparable in all three treatment modalities [60].

IFX has been shown to significantly improve the BCVA [56], lower the frequency of ocular attacks [67,68,69] and retinal vasculitis [66,69], and furthermore reduce the central macular thickness in patients with severe posterior uveitis [4]. Nevertheless, IFX seems to have no advantage over conventional immunosuppressive therapies in preventing macular complications [69,70]. Initiating IFX therapy within the first 18 months of the uveoretinitis onset is more efficient in preserving the BCVA than after 18 months. For this reason, it is advised to switch soon after admission if the patient presents with a severe ocular involvement [71].

Yalcindag et al. recommend IFX as a first line agent in treating acute sight-threatening uveitis [4]. Horiguchi et al. did not reveal any difference between IFX monotherapy and combination therapy in their 10-year follow-up study [68]. They concluded, however, that a high study retention rate after six years of the treatment may be caused by the development of the anti-IFX antibodies, followed by a secondary inefficacy of IFX after six to ten years, which should be taken into consideration in a long-term therapy [4,68]. IFX has a rapid onset of action in comparison to the IFN-alpha [7].

ADA is the first and only non-corticosteroid agent approved by the Food and Drug Administration in the treatment of noninfectious intermediate, posterior, and panuveitis [61]. ADA is claimed to be the best immunomodulatory drug that efficiently controls noninfectious uveitis over the long term [61]. ADA (40 mg s.c. every other week) is believed to preserve visual acuity [7,63,66], reduce the central macular thickness (CMT) and decrease the activity of retinal vasculitis [63]. In a study by Fabiani et al., 64% of the patients presented a resolution of retinal vasculitis in FA after three months of therapy, and 95% were free of active vasculitis after 12 months [63]. The authors state that their findings support the use of anti-TNF agents as the first-line therapy in severe BU [63], which stands in line with the recommendation of American Academy of Ophthalmology [65]. Combination therapy with ADA and immunosuppressive agents seems to have no advantage over ADA monotherapy [63]. ADA appears to induce better improvement of BCVA, vitritis and anterior chamber inflammation than IFX after one year of treatment of refractory BU [66].

Anti-TNFs reduce the optic disc and vascular leakage [4,7,9] and enable a gradual reduction of systemic CSs until either discontinuation or low maintenance dose [7,9,63,66]. Patients should be screened for tuberculosis and demyelinating disease prior to the use of ADA and IFX, as those diseases may activate during immunosuppressive treatment [2,4,5,7,64,66]. Alongside an increased risk of infection, the most common adverse effects of the anti-TNFs are mild local reactions to infusions or subcutaneous injections [4].

Interferon alpha (IFN-alpha) is implied to be an effective second-line treatment for refractory and/or recurrent cases [2,7,37,55,56,63,64].

Celiker et al. used an algorithm where IFN-alpha is a second line treatment and anti-TNFs are used in case of IFN-alpha inefficacy [5]. In their study, 41.3% of patients had to be treated with biological agents to prevent vision loss. [5] The failure of one anti-TNF does not exclude a successful response to another anti-TNF [5,63].

IFN-alpha is composed of several proteins extracted from a leukocyte fraction of human blood that have immunomodulating properties. It is considered a second line treatment due to its side effects and costs; however, it has been proven effective in treating mid-term and long-term ocular manifestations [11,48,56,64]. IFN-alpha is reported in the literature to be administered subcutaneously in a dose of 3–18 million units, 3–7 times per week [4,7,11,53,64,71], achieving a response rate from 75% to 92% [4,53,64]. It is efficient within two to four weeks after initiation [11]. Shi et al. reported a significant decrease of the relapse rate to 0 per patient/year in a mean follow-up time of 21.7 ± 7.5 months after the following treatment: 3 million units daily for four weeks, then 3 million units every other day for three to four months, further tailored according to response [64]. Yalcindag et al. have administered 3 million units in female and low body weight patients and 4.5 million units in others. They increased the dose to 6 million units in recurrent or recalcitrant cases. BCVA improved in 87.8% of the cases [4]. Diwo et al. reported a reaction to IFN-alpha in 86.1% of the patients with severe posterior uveitis in their study, whereas 69.4% never relapsed [11]. Their study revealed a steady decrease of the CME after initiation of IFN-alpha therapy during the first four years of treatment, with no relapses for nine years overall [11].

IFN-alpha is potentially beneficial for refractory neuro-BD, which makes it a reasonable choice for the ocular and parenchymal neurological phenotype [42]. It reduces optic disc and vascular leakage, relapse frequency, anterior segment inflammation, vitreous haze, retinal vasculitis and retinitis [4,11]. A long remission rate, even after discontinuation, seems to be an advantage of IFN alpha therapy over anti-TNF [11]. The most common side effects of IFN-alpha are flu-like symptoms (88–100%) [5,11,53,64], fatigue [11], myalgia [11], leucopenia [53,64] and depression [5,11,53]. The flu-like symptoms are a sign of the absence of the anti-IFN antibodies [4]. Four to eight percent of patients discontinue treatment due to side effects [11]. IFN retinopathy is formally impossible to differentiate from Behçet’s infiltrates [11].

In the study of Yalcindag et al., no difference between the outcomes of treatment with IFX and IFN-alpha was revealed—both were proven to equally control ocular inflammation [4]. Interestingly, patients that were non-responders to IFN-alpha did not have any uveitis relapses after switching to IFX [4].

An alternative use of other biologics has been reported that needs further investigation: tacrolimus [7], tocilizumab [37] (i.v. 8 mg/kg/4 weeks) [7], golimumab [7], anakinra (s.c. 100 mg/daily) [7], canakinumab (s.c. 150 mg/6 weeks) [7] and rituximab [56]. Daclizumab was shown to be ineffective in BU [56]. A high withdrawal rate from the studies using gevokizumab may suggest its inefficacy, but this needs further validation [56].

## 4. Discussion

The etiopathogenesis of BD continues to be understood as a heterogenous concept, with several novel divisions reported in the literature. Shahram et al. suggest a separate pathway of development for ocular and extra-ocular types of BD after analyzing symptom patterns among different ethnic groups [14], while Hussein et al. pointed out a possibility of separate immunological variants with central and peripheral impact based on the higher severity of ocular manifestations in patients who fulfill fewer systemic criteria of BD [21]. Bettiol et al. have correlated parenchymal neurological and ocular symptoms based on literature research and have proposed to consider those patients as one phenotype and therefore adjust the treatment algorithm [42]. Nevertheless, another study on a Chinese cohort divided BD differently without connecting ocular and neurological BD into one cluster [52]. Further investigation of the various phenotypes of BD is recommended to revise the previous concept of BD management. Researchers suggest that the treatment of BD requires a multidisciplinary approach and should be planned according to the correlated groups of symptoms which will improve the general outcome, instead of focusing on each manifestation separately [24,42,52].

An early diagnosis is necessary to prevent permanent vision loss, as BU responds to a specific immunosuppressive algorithm. SD OCT, EDI OCT, OCTA, PNFL OCT and FA enable the detection of CME and retinal neovascularization, as well as the subclinical inflammatory process in patients with systemic symptoms characteristic of BD and, hence, the introduction of appropriate medication [2,6,28,31,32,34,35,36]. UWF captions of color photography and FA contribute to better assessment of necessary treatment adjustments [37]. Laser flare-cell photometry has been suggested to be a reliable tool in the monitoring of ocular inflammation in BU [38,39]. Even though FA remains the gold standard in terms of the monitoring of ocular inflammation in BU, the abovementioned noninvasive diagnostic tools may enable a decrease in the number of FA acquisitions and, moreover, may provide objective, quantitative indices of ocular inflammation. CMT, MV [28] and choroidal thickness [34] were reported to increase during an active inflammation. CMT has been reported to decrease below the normal values in the remission periods, which was significantly correlated to the number of uveitis attacks, which can be an indicator of a permanent damage to the retina [28]. Furthermore, CMT and MV have reduced in response to treatment in active uveitis, which supports the role of SD OCT as a monitoring tool [28]. Chams et al. revealed a fluorescein leakage in the FA in 44% of their patients with BD with no signs of ocular inflammation, indicating the value of FA for early detection of retinal vasculitis [31].

Behçet’s disease ocular attack score 24 [47] and total vascular leakage score [29] have been newly proposed as effective tools to facilitate the evaluation of BD along the course of the disease. Inhomogeneity of the criteria used to describe the activity of BD among study groups seems to be a limitation to this review. Development of an objective and universal scale of BD activity would facilitate the comparison of results between physicians, and therefore further investigations seem advisable.

AZA has been long considered the first line agent, followed by CSs, ADA, IFX, IFN-alpha and CYC in variations according to the disease activity and reaction to therapy [4,7,11,42,55,56,63,64,66,67,68,69]; however, the role of immunomodulatory therapy has grown, due to the clinical evidence of its efficacy [60,64,65,66,67,68,69,70,71]. The American Academy of Ophthalmology has strongly recommended IFX and ADA as both first and second line of corticosteroid-sparing therapy of BU [65], which stands in line with findings of other researchers [60,61,62,63,64,68]. CSs should be always accompanied by other immunosuppressive agents to achieve the steroid-sparing effect [2,7,25,27,54,55,56]. Intravitreal and periocular CS injections [14,55] and dexamethasone intravitreal implants [30] are additional investigated options of local treatment. The indications to the use of CsA in BU should be reviewed due to the suggested coexistence of ocular and neurological manifestations and the neurological side-effects of CsA [37,57,58], which is in contrast to The European Alliance of Associations for Rheumatology recommendations from 2018 [55]. A limitation to this study is the inclusion of both RCTs and unrandomized clinical studies, leading to a lower reliability of the analyzed diagnostic and therapeutic solutions. There is no strict consensus on the treatment of the ocular BD, and for this reason there is a great need for randomized controlled trials to objectively compare treatment algorithms.

## Figures and Tables

**Table 1:** Epidemiology of Behçet’s disease.

Region	Prevalence Rate per 100,000 Inhabitants
Turkey	20–420 [13]
Iran	80–100 [14]
Northern Europe	0.3–4.9 [13]
Sweden	2.3–4.9 [13]
Germany	0.3–4.87 [13,15]
England	0.27–0.64 [13]
Southern Europe	1.5–15.9 [2,13]
Italy	3.8–15.9 [13]
Spain	7.5 [13]
France	7.1 [13]
Portugal	1.53 [13]
United States	5.2 [14]

**Table 2:** Differential diagnosis of Behçet’s disease ocular manifestations.

Differential Diagnosis	Characteristic Features
Sarcoidosis	Intermediate uveitis, snowballs, focal retinal leakage, choroidal nodules, iris nodules, optic disc nodules, mutton-fat keratic precipitates, segmental/nodular periphlebitis (candle-like drippings) [48]
Tuberculosis	Usually associated with anterior segment inflammation, peripheral ischemia, snowballs, perivascular choroidal scars, broad-based posterior synechiae, serpiginous-like choroiditis [9,49]
Syphilis	Iritis, iridocyclitis, wedge shaped, ground-glass retinitis with inner retinal precipitates, chorioretinitis, placoid lesions at the level of retinal pigmented epithelium [50]
Viral retinitis	Massive necrotizing retinitis, CMV: absence of intense vitreous haze with severe retinitis [9]
Toxoplasmosis	Granulomatous keratic precipitates, hypertensive anterior uveitis [9]
HLA-B27-associated acute anterior uveitis	Slow response to topical steroids, fibrinous exudate, sticky hypopyon [9]
Primary intraocular lymphoma and leukemia	Smooth-layered hypopyon with mild ciliary injection [9]

## Data Availability

Not applicable.
